# Role of surveillance data in detecting malaria outbreaks in an epidemic-prone region in Kenya: findings from an investigation of a suspected outbreak in Nandi County

**DOI:** 10.1186/s12936-024-05216-2

**Published:** 2024-12-18

**Authors:** Geoffrey Kongo Githinji, Fredrick Ouma Odhiambo, Clara Muyaku Andala, Daniel Chepkwony, James Kibet Sang, Maurice Owiny, Japhet Ruto, Elvis Omondi Oyugi, Fredrick Odhiambo

**Affiliations:** 1 Kakamega County Department of Health, Kakamega, Kenya; 2 Field Epidemiology and Laboratory Training Programme, Ministry of Health, Nairobi, Kenya; 3 Kisumu County Department of Health, Kisumu, Kenya; 4 Taita Taveta County Department of Health, Voi, Kenya; 5 Nandi County Department of Veterinary Services, Kapsabet, Kenya; 6 Division of National Malaria Programme, Ministry of Health, Nairobi, Kenya; 7 Nandi County Department of Health, Kapsabet, Kenya

**Keywords:** Kenya, Malaria, Outbreak, Data accuracy

## Abstract

**Background:**

Approximately 70% of the Kenyan population is at risk for malaria, including 19 million people in highland epidemic-prone and seasonal transmission areas. Surveillance data showed a 288% increase in malaria cases and an incidence rate of 10.5 per 1000 population between January and May 2021 in Nandi County. We investigated the increased incidence of malaria in Nandi County.

**Methods:**

We abstracted demographic and clinical data from the laboratory register in health facilities with high malaria burden. Key informant interviews using a structured questionnaire collected healthcare worker perceptions on malaria interventions and personnel capacity. We calculated means and medians for continuous variables and frequency and proportions for categorical variables. Data quality assessment (DQA) was conducted to evaluate timeliness and completeness, data accuracy, and overall system assessment.

**Results:**

We reviewed 19,526 records from 12 health facilities. Females contributed 61% cases (11,862). A majority of cases, 21% (4111), were between the age group 15–24 years. Of the 19,498 tested, 2662 tested positive (test positivity rate, TPR = 13.7%). Microscopy accounted for 39% (1041) and RDT for 61% (1620) of tests conducted, with some patients being double tested using both tests. Kapsabet County Referral contributed 26% (5051) suspected cases, TPR 3.2%, and Chemase Health Centre TPR was 33.2%. Facilities experienced major RDTs stock-outs in the preceding 3 months while three (30%) of the 10 facilities assessed conducted laboratory Internal Quality Control (IQC) programmes. Of the 12 facilities assessed, four (33%) facilities had an over-reporting of suspected cases in the monthly summary, while three (25%) facilities were over-reported in the online tool. On reporting confirmed malaria cases, over-reporting was noted in three (25%) facilities in both the monthly summaries and the online tool. Data completeness was 77% and timeliness 93%.

**Conclusion:**

The increase in malaria cases in Nandi County displayed a seasonal pattern that coincided with either the long or short rainy seasons, the investigation did not reveal an active outbreak at the time of the inquiry. Sub-county hospitals in Tinderet and Aldai sub-counties had malaria cases exceeding both the alert and action thresholds at specific times during the year under review, suggesting a potential occurrence of unidentified outbreaks, while several other facilities had an increase of cases reaching alert thresholds, indicating upsurges. In healthcare settings, we noted there were problems with data quality. We advised routine data review, analysis, and feedback; mentorships for data analysis and on the job and support supervision; mentorships for malaria diagnosis; and installation of laboratory quality assurance.

## Background

In epidemic-prone regions such as Nandi County, Kenya, where malaria remains a significant public health concern, the timely detection and effective response to outbreaks are crucial for minimizing morbidity and mortality. The Division of National Malaria Programme, through the Kenya Malaria Strategy 2019–2023, seeks to protect 100% of people living in malaria risk areas through access to appropriate malaria interventions, manage 100% of suspected malaria cases according to Kenya Malaria Treatment Guidelines, and strengthen malaria surveillance and use of information to improve decision-making. Thus, rapid response to these epidemics can be made where effective surveillance systems are in place for early recognition of disease incidence anomalies [1]. Epidemics are associated with unusual climatic conditions, mainly high rainfall and temperatures suitable for vector breeding [2].

Epidemic-prone counties are particularly vulnerable to recurring outbreaks due to the presence of favorable conditions for the proliferation of malaria vectors. Malaria epidemics in Kenya usually affect western highlands, arid and semi-arid regions [2]. Epidemic-prone counties are regions characterized by a high risk of disease outbreaks and often susceptible to the rapid spread of infectious diseases due to various factors, including environmental conditions. Nandi County is one such region with the county's geographical features, including its warm climate, abundant rainfall, and suitable breeding sites for mosquitoes, creating an environment conducive for the transmission of malaria parasites. More recent malaria epidemics have been reported in epidemic prone counties of Turkana, Baringo, Isiolo, Mandera, Marsabit, and other neighbouring counties [2], with malaria risk maps modelled from countrywide survey data [3] showing evidence of changing malaria epidemiology with more areas likely to become unstable and prone to epidemics.

Effective surveillance systems play a pivotal role by providing critical data for monitoring disease trends, identifying outbreaks, identifying emerging hotspots and guiding targeted interventions. Reliability and quality of surveillance data are essential factors that determine the effectiveness of these systems. Poor data quality, including inaccuracies, delays, and incompleteness, can impede the early detection and response to malaria outbreaks, hampering the implementation of appropriate control measures. The quality of routine data generated, and diagnostic tests performed could impact the strength of malaria surveillance systems. Health facility data is the primary source for assessing health sector performance. The quality of routine data and diagnostic tests reported by health facilities should be regularly evaluated, and required investments should be made to ensure the data is reliable and useable using standardized assessment tools like the Malaria Routine Data Quality Assessment tool (MrDQA) and laboratory external quality assessments. Case studies from countries that have successfully eliminated malaria have shown that good quality routine data are needed to evaluate the technical, operational, and financial feasibility of achieving malaria elimination [4].

Malaria epidemics and outbreaks had previously been reported within Nandi County, with previous studies indicating that malaria transmission occurs in “hot spots” and is seasonal and sporadic [5, 6]. Other investigation studies in the Nandi region have shown spatial clustering of malaria cases in children during an epidemic [7]. The Highland Epidemic Prone Malaria zone, which includes Nandi County and fourteen other counties, has a population of nearly seven million at risk of malaria infection residing in these areas translating into approximately 23% of the total country population [8, 9]. Tinderet and Aldai sub-counties have been shown to have pockets of focal increased transmission intensities exceeding the average levels i.e. “Hotspots” in previous studies [10],with altitude and temperatures shown to be the vital factors affecting the development and survival of vectors [7]. The sub-counties also border the all-year stable malaria lake endemic region of Kisumu and Kakamega counties. The top 10 causes of morbidity and mortality in Nandi County contribute to about 90% of disease cases and about 40% of deaths. Upper Respiratory Tract infections with a prevalence rate of 44% and malaria with a prevalence of 21% are the two leading causes of morbidity and mortality in Nandi County [11]

**Fig. 1:**
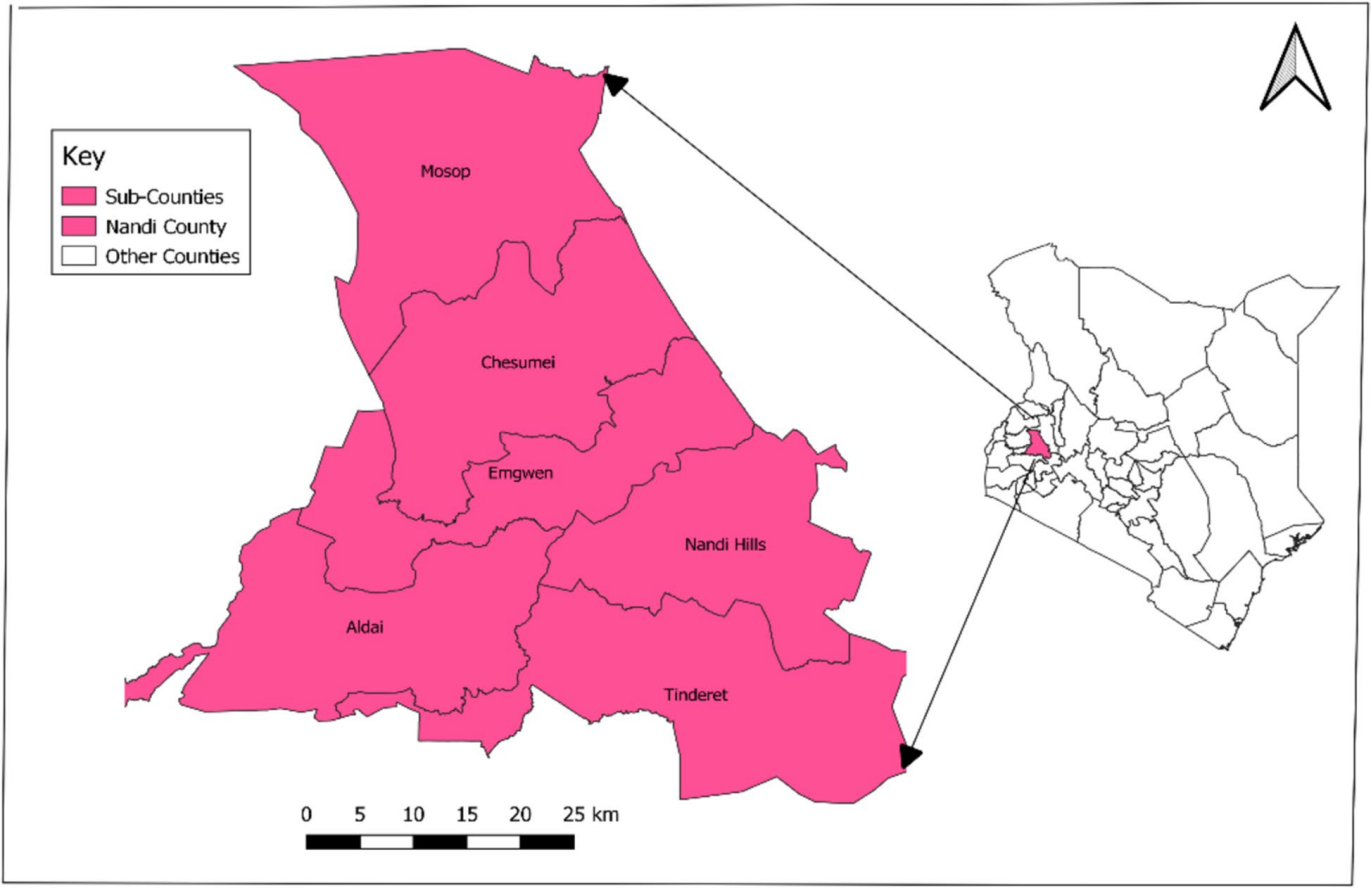
An administrative map of Nandi County

Nandi County (Fig. 1) reported an increase in total confirmed cases from January 2021 to May 2021, as represented by the data sourced from Kenya Health Information System (KHIS).

According to the outpatient monthly reports (MOH 705 A and B) on KHIS, there were 1284 confirmed malaria cases in January 2021, while in May 2021, the number almost quadrupled to 4985, a 70% increase compared to May 2020. Aldai and Tinderet were the most affected sub counties, with the highest no of total confirmed cases showing a sharp increase in Malaria cases from Jan 2021 to May 2021, following a decline from June 2020 to Dec 2020. The other sub-counties show a slight increase in the number of confirmed total malaria cases from February to May 2021.

A literature review for recent research on malaria data in Nandi County reveals little information on trends in malaria surveillance, whilst data from KHIS shows an upward trajectory of malaria cases in Nandi County. The average distance to the county's nearest health facility is seven kilometers compared to the required maximum of three [11]. Recent malaria risk profile for Kenya indicates the population of Nandi having a risk of between 1–5% PA*Pf*PR_2–10._ The county reported a malaria incidence of 44.7 cases per 1000 population in 2020 with 35,773 cases reported in the same period. Tinderet and Aldai sub-counties were the most affected, both reporting an incidence of between 40–< 100 cases per 1000 population [12]. Malaria epidemics are most likely to be frequent and intense if the current intervention strategies—accurate case management of malaria according to the national guidelines, targeted vector control with LLINs coverage, IRS and LSM, surveillance and monitoring activities, epidemic preparedness response activities and targeted SBC messaging—are not strengthened and sustained. Such epidemics would strain the healthcare system due to increased health facility visits and hospital admissions as well as the need for commodities, human resources for health, and other resources and hinder the overall goal for the Division of National Malaria Programme to reduce malaria incidence and deaths by at least 75% of the 2016 levels by 2023. Success in controlling and reducing malaria incidence cases and deaths in Nandi County depends on reliable surveillance, monitoring, and evaluation. The investigation reported on the assessment of the increased malaria incidence, the quality of data and the quality of malaria diagnostic tests in Nandi County.

## Methods

### Study sites

We conducted the investigation in Nandi County, located between latitude 0034N and longitude 34045E to the west while the eastern boundary reaches Longitude 35025E. It covers an area of 2884.4 km^2^; and borders Kakamega County to the West, Uasin Gishu County to the Northeast, Kericho County to the Southeast, Kisumu County to the South, and Vihiga County to the Southwest. The northern parts of the county receive rainfall ranging from 1300 to 1600 mm per annum, while the southern half receives rainfall as high as 2000 mm per annum due to the influence of Lake Basin atmospheric conditions. Most parts of the county experience mean temperatures ranging between 18 to 22 °C during the rainy season; but the part adjacent to the Nyando escarpment at 1300 m above sea level, experiences average temperatures as high as 26 °C [11].

### Investigation period

We conducted the investigation from June 29, 2021, through July 12, 2021.

### Investigation population

Our investigation population consisted of records review in Nandi County. We visited twelve facilities, and their selection was based on sentinel surveillance sites used by the county to monitor malaria incidence in the county and had a high malaria caseload.

### Investigation design

We used a mixed-method approach; Quantitative methods entailed a retrospective review of suspected malaria cases in health facility records, while qualitative methods involved key informant interviews with healthcare workers using a semi structured questionnaire.

Key informant participants were purposively selected to represent different cadres and malaria service delivery departments. They included laboratory officers, public health officers, nurses, clinical officers, and pharmaceutical technologists.

We conducted malaria data quality assessment and Laboratory External Quality Assurance (EQA) in selected health facilities in the county using the revised digital malaria routine Data Quality Assessment (MrDQA) and Laboratory EQA assessment tools. A suspected malaria case was defined as an entry into the health facility records in Nandi County during the review period, presenting with fever and/or exhibiting any of the following symptoms: headache, backache, chills, sweat, myalgia, nausea, and vomiting. This definition aimed to capture individuals who showed signs indicative of malaria infection. A confirmed malaria case was defined as any entry in the health facility records in Nandi County during the review period, who had been officially confirmed as having malaria through a positive blood smear or a positive malaria rapid diagnostic test (RDT).

### Data collection, processing and analysis

Key informant interviews collected information on healthcare worker perceptions of malaria interventions, prevention practices, case management and personnel capacity. Information captured included participants years of experience, familiarity with national guidelines and strategies, training history in malaria-related competencies, and experience with malaria data collection tools. Other areas assessed included surveillance practices, malaria intervention implementation, commodity management and data review processes. The responses were analyzed using descriptive statistics for quantitative data and thematically for the open-ended questions to identify key challenges and practices in malaria control and prevention.

For data collection on characteristics of suspected malaria cases in the outpatient department, a spreadsheet data abstraction tool was utilized to abstract data from health record registers between January 2021 and June 2021. The variables included patients' socio-demographic information (age, sex, residence) and clinical features (date of onset, date of visit to the health facility, signs and symptoms, diagnosis, treatment, comorbidities, and patient outcome). We calculated means and medians for continuous variables, and frequencies and proportions for categorical variables, while facility test positivity rates were presented using maps. Malaria data quality assessment in selected health facilities was done using the revised digital malaria routine Data Quality Assessment (MrDQA) tool. The data sources for assessment included facility outpatient registers (MOH 204 A and B), facility monthly summary (MOH 705 A and B), Facility Laboratory Monthly Summary (MOH 706), Facility Laboratory Registers (MOH 240), Malaria commodities monthly summary tool (MOH 743), and the malaria commodities Daily Activity Register—DAR (MOH 645).

The tool comprised standardized checklists that evaluated malaria and programme data in several ways: First, the timeliness and completeness of data elements were evaluated by reviewing the last three monthly summary reports (MOH 705 A or B) to check if they were submitted by the reporting deadline. The proportion of reports submitted on time was calculated to assess compliance with the national malaria programme's recommendations. Data element completeness was assessed by counting the number of entries with missing information for each data element, based on the source document for the corresponding period. Reporting accuracy was assessed by comparing validated values from the source register (outpatient register—MOH 204 A or B) with the reported values from the monthly report (monthly summary—MOH 705 A or B and data in the electronic Kenya Health Information System-KHIS) to derive the verification factor (VF). Discrepancies between validated and reported values were investigated, and VF values below 0.9 (90%) or above 1.1 (110%) were indicative of over-reporting or under-reporting, respectively.

Cross-checks were conducted by comparing a random sample of ten records from the primary facility data source (Outpatient register—MOH 204 A or B) with a matching record from a second primary facility data source (Laboratory or Daily Activity Register—MOH 240 or MOH 645) to assess matching information for priority data fields. The percentage of matching records over the sample size compared was calculated as an indicator of data quality, with values of 90 percent or higher considered acceptable.

To evaluate the consistency of reported data over time, data from the last three consecutive months and the current month were compared to the same month from the previous year. This comparison aimed to assess the plausibility of current results in relation to historical precedents, with minimal changes expected in the comparison indicator unless there were significant demographic changes in the facility's catchment area. A system assessment checklist of best practices for producing good quality data was employed to evaluate the ability to ensure data quality. The checklist consisted of twelve questions, with investigators noting "yes" or "no" based on the presence or absence of specific practices at the facility, respectively. For the assessment of laboratory external quality assurance processes (EQA), data were collected using an EQA laboratory checklist tool. This tool captured variables related to facility information, laboratory staffing, staff training and experience, commodities and supplies, internal and external quality assurance, test results turnaround time, laboratory reference materials, and laboratory information system. The resulting categorical variables were summarized using frequencies and proportions.

## Results

### Suspected outpatient malaria cases

**Fig. 2:**
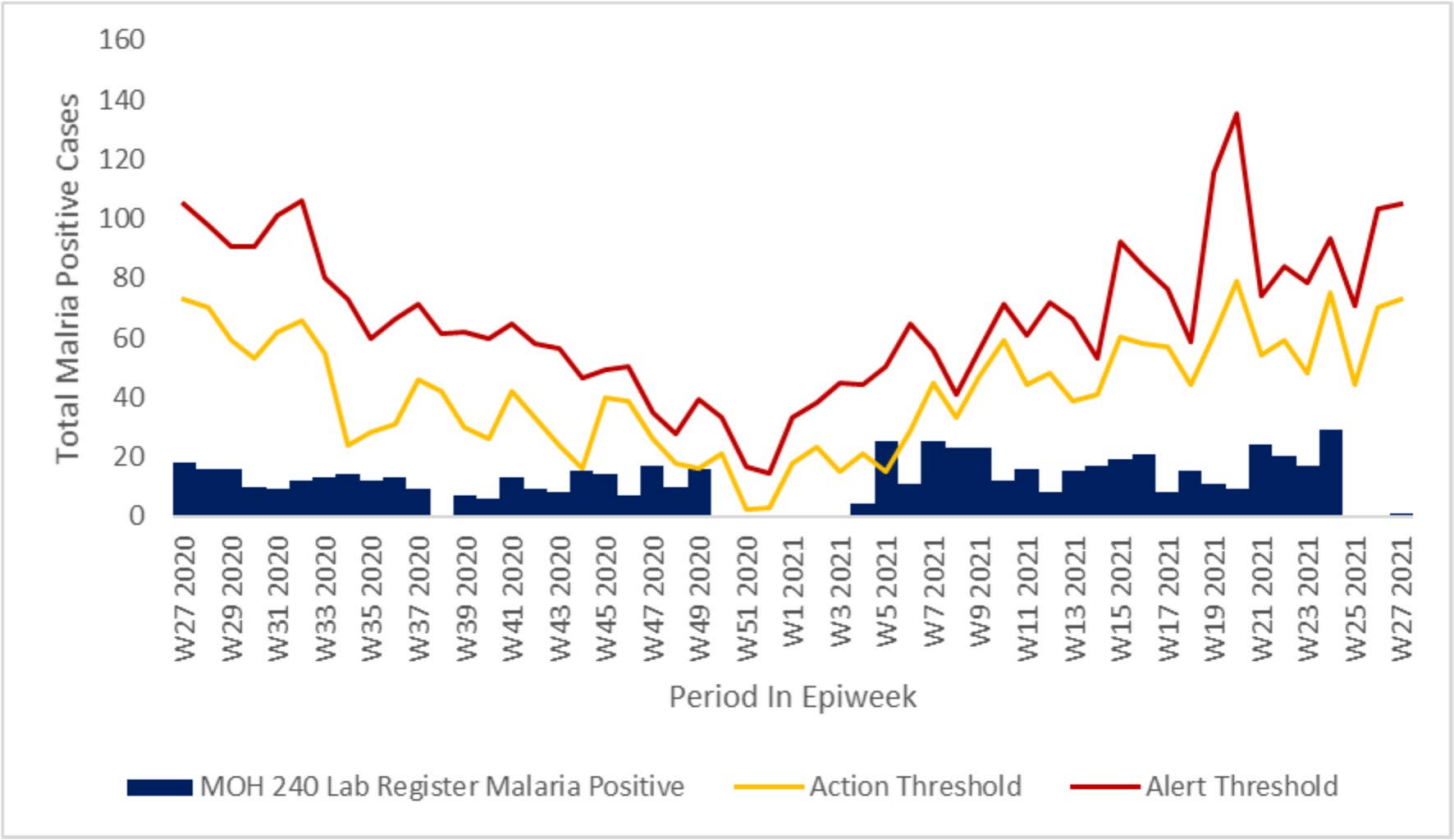
Chemelil (Poto Poto) dispensary epicurve, July 2020–June 2021

**Fig. 3:**
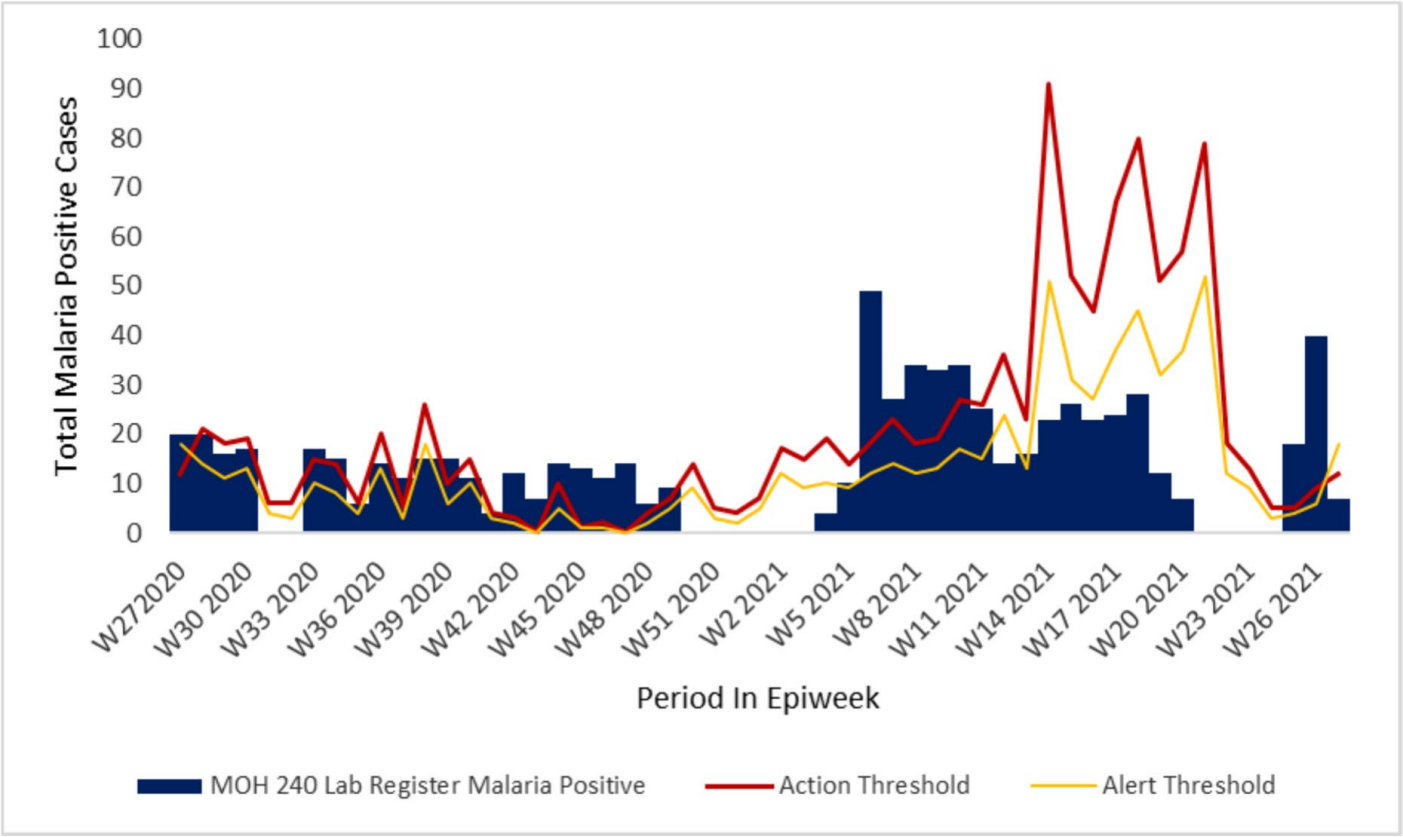
Meteitei sub-county hospital epicurve, July 2020–June 2021

**Fig. 4:**
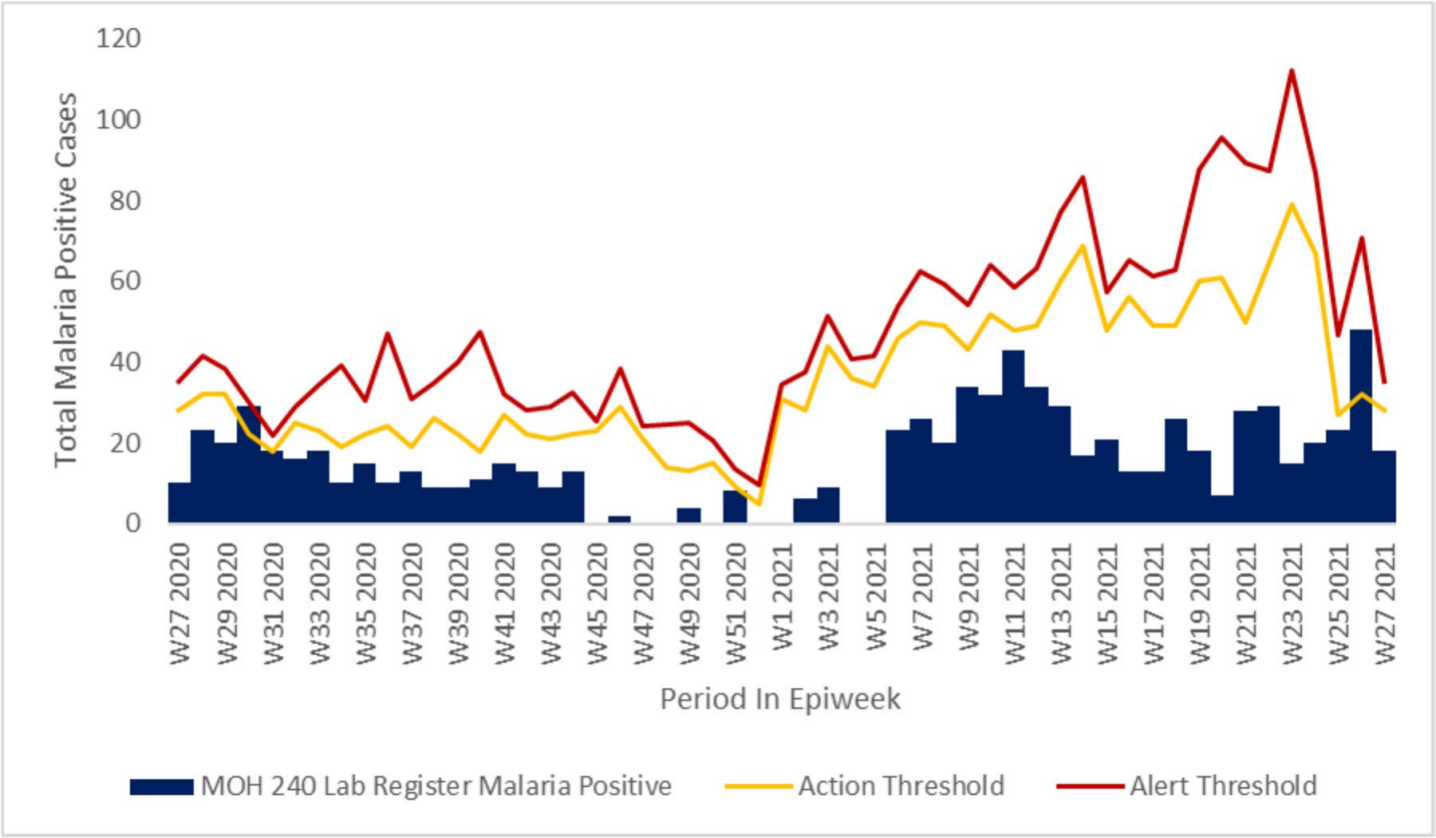
Chemase Health Centre epicurve, July 2020–June 2021

Epidemic curve across all the facilities reviewed indicated increased incidence of malaria cases from epi week twenty-one through epi week twenty-seven of the investigation period in accordance with the seasonality pattern seen across the year in review, with some of the facilities reviewed having cases reaching alert threshold levels but never getting to action threshold levels in Chemelil health centre (Fig. 2). In Chemase and Meteitei sub-county hospitals we noted malaria cases surpassed the alert and action thresholds during certain durations of the year in review indicating a possible outbreaks occurrence. We also noted that the county and sub-county surveillance teams did not report on these surpassed thresholds or conduct investigations (Figs. 3 and 4).

**Fig. 5:**
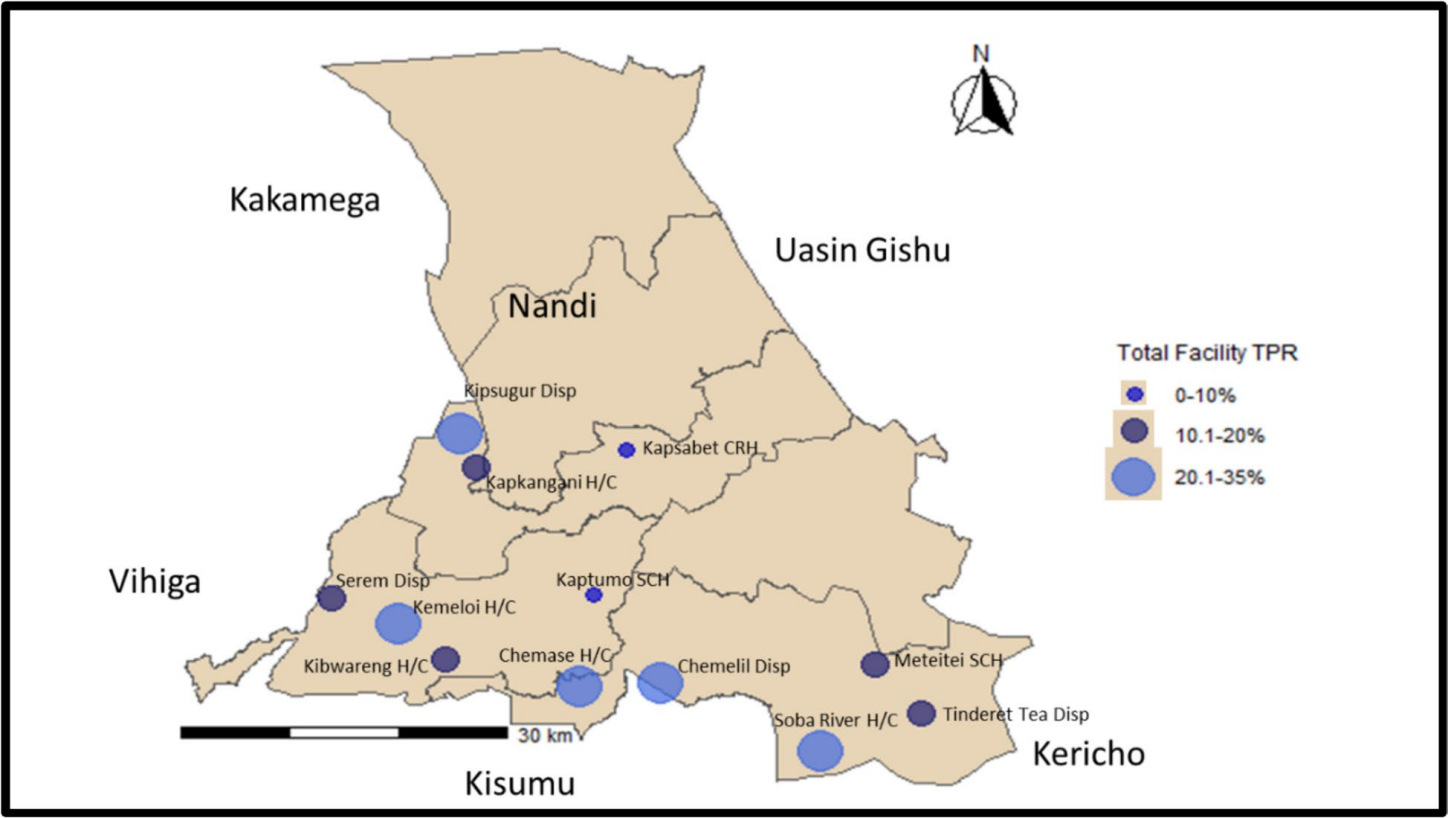
Total Positivity rates per facility for cases abstracted in Nandi County, April–June 2021

We reviewed 19,526 records of suspected malaria cases 12 health facilities; The median age was 19 years (Interquartile Range, IQR = 29). Females contributed 11,862 (60.7%), the 15–24 years’ age group contributed 4111 (21.1%), while the 60 years and above age group contributed 1503 (7.7%). A total of 19,498 malaria diagnostic tests were conducted, 16,836 tested negative, while 2662 tested positive. 11,579 (59.3%) of the tests were diagnosed using microscopy, while 7945 (40.7%) were conducted using RDTs. Of the 2662 confirmed malaria cases 1041 (39.1%) were done using microscopy with a Microscopy Test Positivity Rate (Microscopy TPR) of 9.0% and 1620 (60.9%) were done using RDT (RDT TPR 20.4%) as summarized in Table 1. Chemase Health Centre had the highest facility test positivity rate (TPR) at 33.2% despite testing fewer patients, 879 (4.5%) cases of total reviewed records (Fig. 5). Kapsabet County Referral Hospital contributed the highest number of cases, 5051 (25.9%) with a facility TPR of 3.2%. Tinderet Tea Dispensary contributed to the least number of cases, 204 (1.04%) with a facility overall TPR of 12.3% (Fig. 5). The overall test positivity rate (TPR) for all the facilities assessed was 13.7%.

**Table 1:** Characteristics of suspected malaria cases in Nandi County in April–June 2021

Variable	Frequency	Percent
*Age group (N = 19,526)*		
< 5	3489	17.9
5–14	4091	20.9
15–24	4111	21.1
25–34	2522	12.9
35–59	3491	17.9
≥ 60	1503	7.7
Not filled	318	1.6
*Sex*		
Female	11,862	61
Male	7647	38.9
Not filled	17	0.1
*Health facility visited*		
Kapsabet CRH	5051	25.9
Kaptumo SCH	2404	12.3
Meteitei SCH	1972	10.1
Kemeloi H/C	1695	8.7
Kapkangani H/C	1450	7.4
Serem H/C	966	5
Chemase H/C	879	4.5
Soba river H/C	568	2.9
Chemelil (Poto Poto) dispensary	390	2
Tinderet tea dispensary	204	1
Kibwareng H/C	2807	14.4
Kipsugur dispensary	1140	5.8
*Type of lab test*		
Microscopy	11,579	59.3
RDTs	7945	40.7
Not filled	2	0.01
*Test results*		
Negative	16,836	86.3
Positive	2662	13.6
Not filled	28	0.1
*Confirmed malaria*		
Microscopy positive	1041	39.1
RDT positive	1620	60.9
Not filled	1	0.04

### Key informant interviews

We interviewed 39 healthcare workers across seven different cadres (Table 2). Most respondents (51.3%) had over 5 years of experience, while 41.0% had 3–5 years of experience. Familiarity with key national malaria documents was limited—only 28.6% had read the 2015 Kenya Malaria Indicator Survey, 25.8% had read the 6th Edition Kenya Malaria Treatment Guidelines, and 34.6% had read the Kenya Malaria Strategy 2019–2023. In regard to capacity building, 48.4% health care workers had received malaria case management training in the last three years, 33.3% had malaria surveillance training, and 30.0% had malaria commodity management training within the same period (Table 3). Among laboratory staff, 75.0% had received malaria microscopy refresher training within the last three years. Major challenges identified included shortage of revised data collection tools, lack of training on new tools, incomplete data entry, and inadequate space in revised registers. Data review meetings were conducted regularly in 75% of facilities, though only 25% of facilities reported analysing malaria data on DHIS2 (Table 3). Healthcare workers primarily used malaria data for monitoring disease trends, epidemic preparedness, community sensitization, and commodity management (Table 3).

**Table 2:** Table of key informant interview participants and their attributes in Nandi County, April–June 2023

Informant	Number interviewed (n)	Department
Laboratory officers	8	Laboratory
Public health officers	4	Community/Sub-County
Nurses	9	Outpatient/ MCH/ Antenatal
Clinical officers	5	Outpatient
Quality assurance officer	1	County Referral Hospital Lab
HRIO	8	Sub-county
Pharmaceutical technologist	4	Pharmacy
Total	39	

Variable	Frequency (n = 39)	Proportion (%)
*Years of experience*		
> 5	20	51.3
3–5	16	41
1–3	3	7.7
< 1	0	
*Have read the 2015 Kenya Malaria Indicator Survey*		
No	20	71.4
Yes	8	28.6
*Have read the 6th Edition Kenya Malaria Treatment Guidelines*		
No	23	74.2
Yes	8	25.8
*Have read the Kenya Malaria Strategy 2019–2023*		
No	17	65.4
Yes	9	34.6

**Table 3:** Table showing health worker malaria training capacity development and health facility capacity in malaria surveillance and case management in Nandi County, April–June 2021

Capacity building programme	Participant response frequency (n)	Proportion (%)
Malaria case management	15/31	48.4
Basic epidemiology	4/31	12.9
Malaria surveillance	10/30	33.3
Malaria commodity management training	9/30	30.0
Malaria microscopy refresher training	6/8	75.0
OJT/mentorship on Artesunate in Managing severe malaria	6/21	28.6

Other areas assessed	Facility response frequency (n = 12)	Proportion (%)
*Surveillance*		
Who captures surveillance data	PHO (4), HRIO (3), Lab Officer (5)	PHO (33.3), HRIO (25), Lab Officer (41.7)
Tool used to capture surveillance data	MOH 505 (8), Other tools (4)	MOH 505 (66.7), Other tools (33.3)
Tools used to summarize surveillance data	MOH 240 (8), MOH 204(4)	MOH 240 (66.6), MOH 204 (33.3)
*Malaria interventions*		
SP/Fansidar stock out in the last 3 months	No (11), Yes (1),	No (91.7), Yes (8.33)
LLINs stock out in the last 3 months	No (9), Yes (3)	No (75), Yes (25)
*Pharmacy*		
AL stock available	Yes (10), No (2)	Yes (83.3), No (16.6)
Artesunate injection available	Yes (8), No (4)	Yes (66.6), No (33.3)
*Data review*		
Conduct facility data review	Monthly (9), Quarterly (2) Weekly (1)	Monthly (75), Quarterly (16.7), Weekly (8.33)
Participate in CMEs on data management	Monthly (7), Quarterly (4) Weekly (1)	Monthly (58.3), Quarterly (33.3), Weekly (8.33)
Analyze malaria data on DHIS	No (9), Yes (3)	No (75), Yes (25)
Is malaria data used for decision making	Yes (9), No (3)	Yes (75), No (25)

### Health facility data quality assessment

**Fig. 6:**
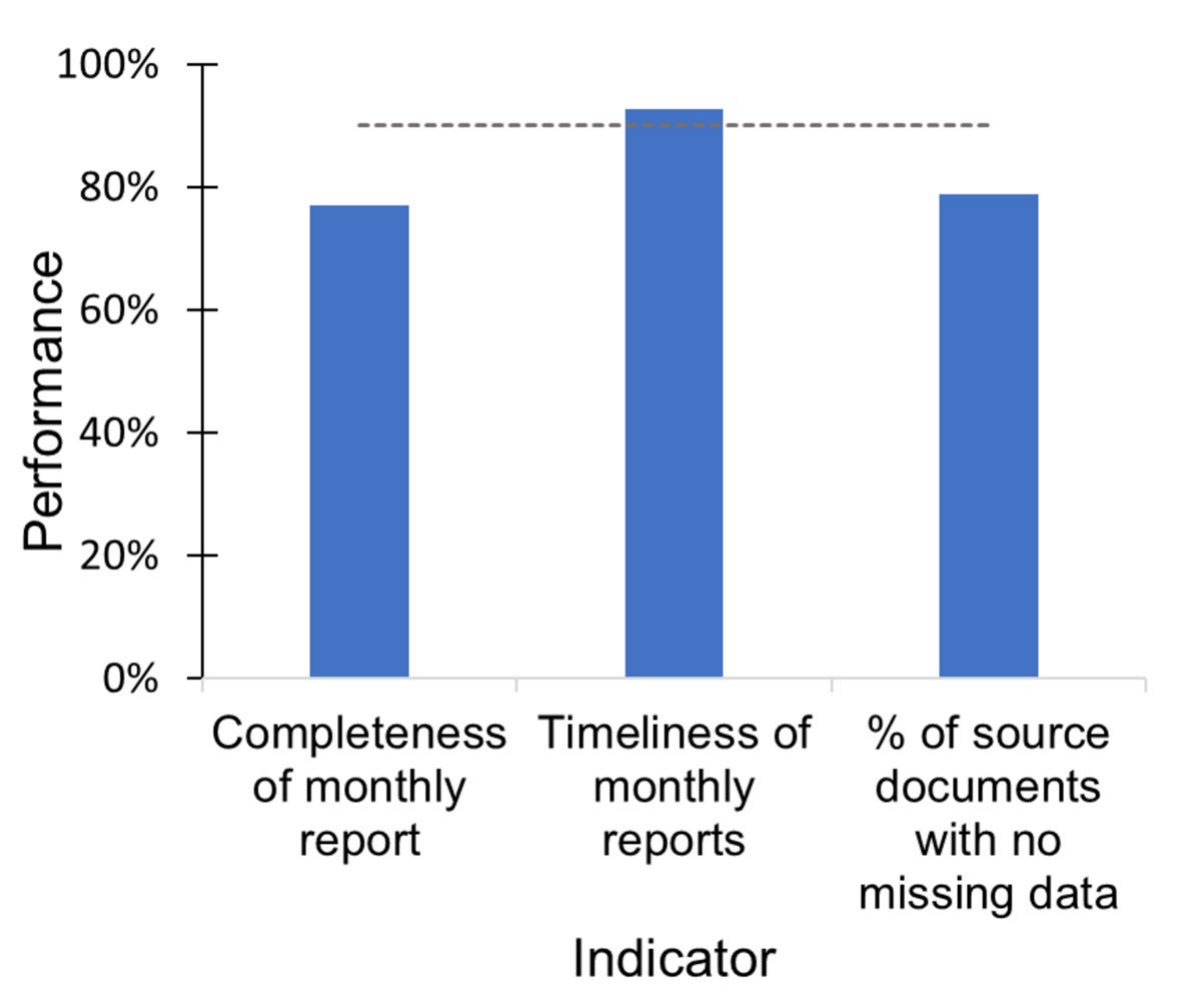
Completeness of source documents and reports with timeliness of monthly reports in Nandi County, March–May 2021

We assessed data quality in 12 health facilities using the electronic routine data quality assessment tool. The overall monthly outpatient summary (MOH 705 A and B), laboratory summary (MOH 706), and malaria commodities summary report timeliness for March to May 2021 was at 93%. The monthly report completeness was at 77%, whereas the outpatient register (primary source document) completeness was at 79% (Fig. 6).

**Fig. 7:**
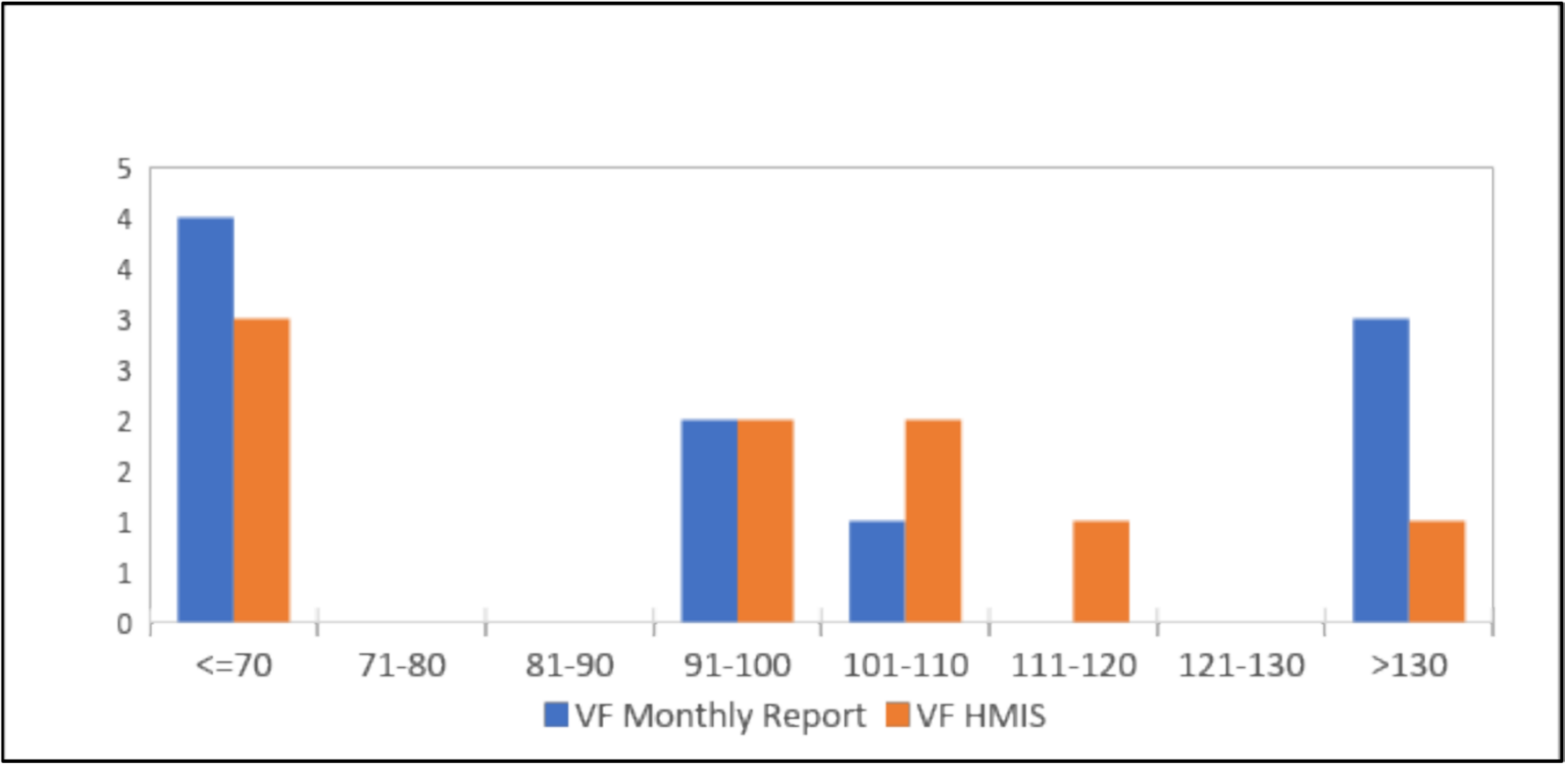
Comparison of the number of suspected malaria cases tested data in Lab Register (MOH 240) versus data reported in monthly summary tools (MOH 705 A and B) and KHIS in Nandi County, March–May 2021 (Quality target: > 75%, < / = 125%). *VF* Verification Factor, *HMIS* Health Management Information System

On the assessment of reporting accuracy, it was noted in reporting of suspected malaria cases, four facilities had a verification factor of less than 70%, indicating over-reporting of cases in the monthly summary report, while three facilities over-reported in the KHIS online tool. Accuracy of monthly reports was recorded in two health facilities (Verification Factor = 100%). Under-reporting by greater than 30 percentage points was noted in three facilities in the monthly summary tool, while one facility under-reported by over 30% in the KHIS (Fig. 7).

**Fig. 8:**
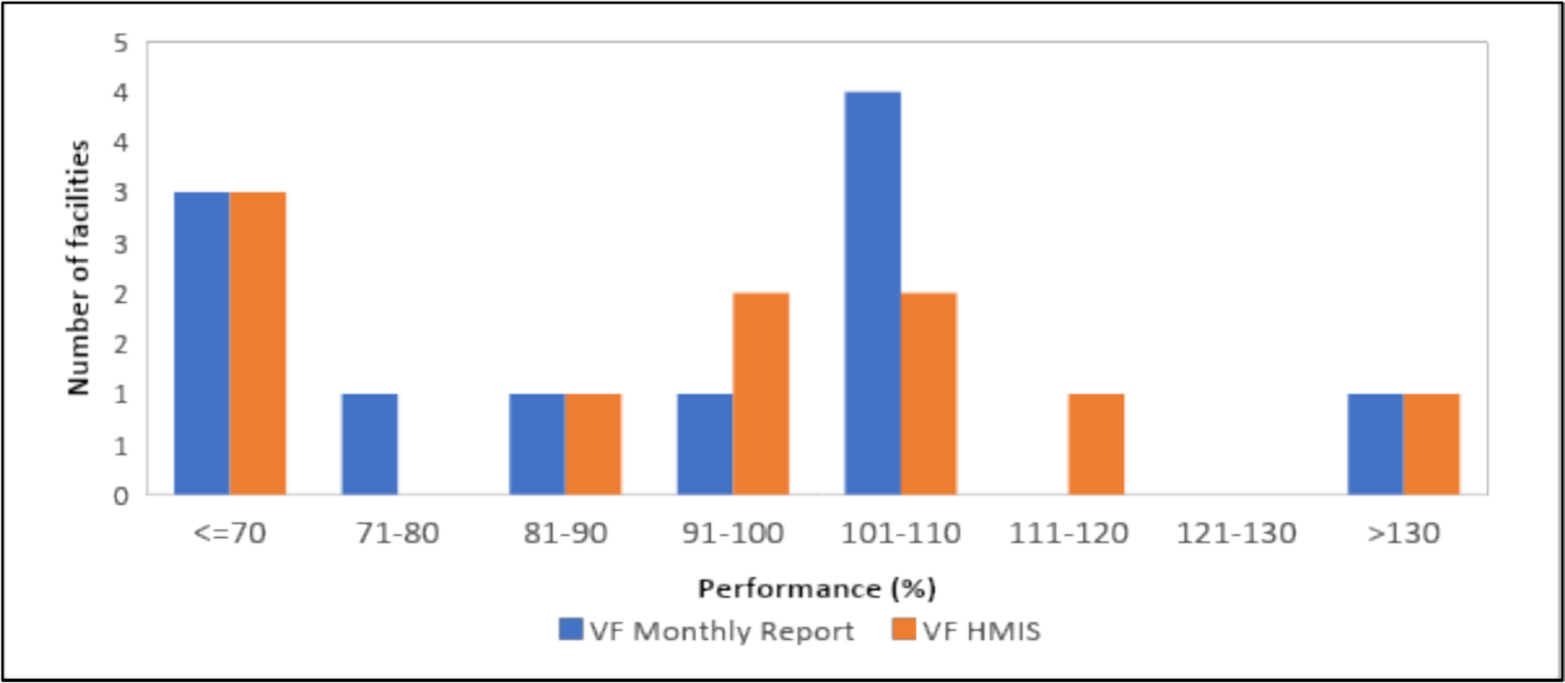
Comparison of number of confirmed malaria cases in Lab Register (MOH 240) versus data reported in monthly summary tool (MOH 705A and B) and KHIS in Nandi County, March–May 2021 (Quality target: > 90%, < / = 110%). *VF* Verification Factor, *HMIS* Health Management Information System

**Fig. 9:**
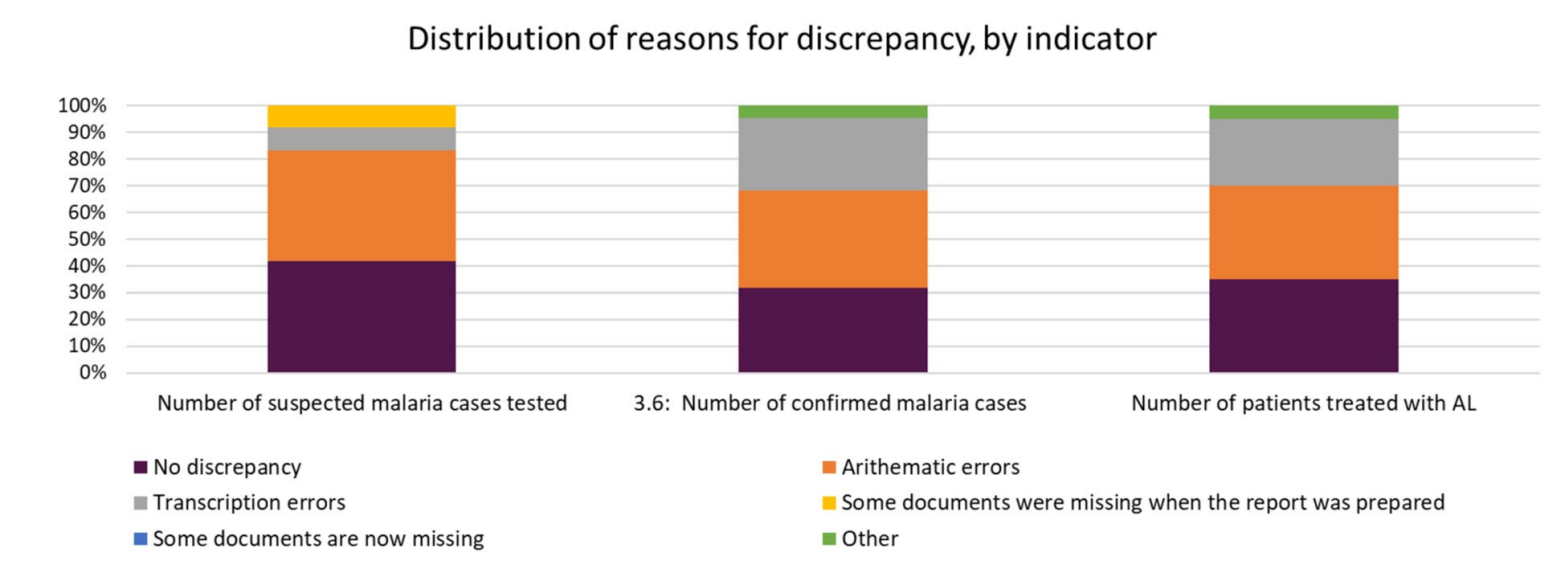
Distribution of reasons for discrepancy, by indicator, in Nandi County, March–May 2021. *AL* Artemether Lumefantrine

**Fig. 10:**
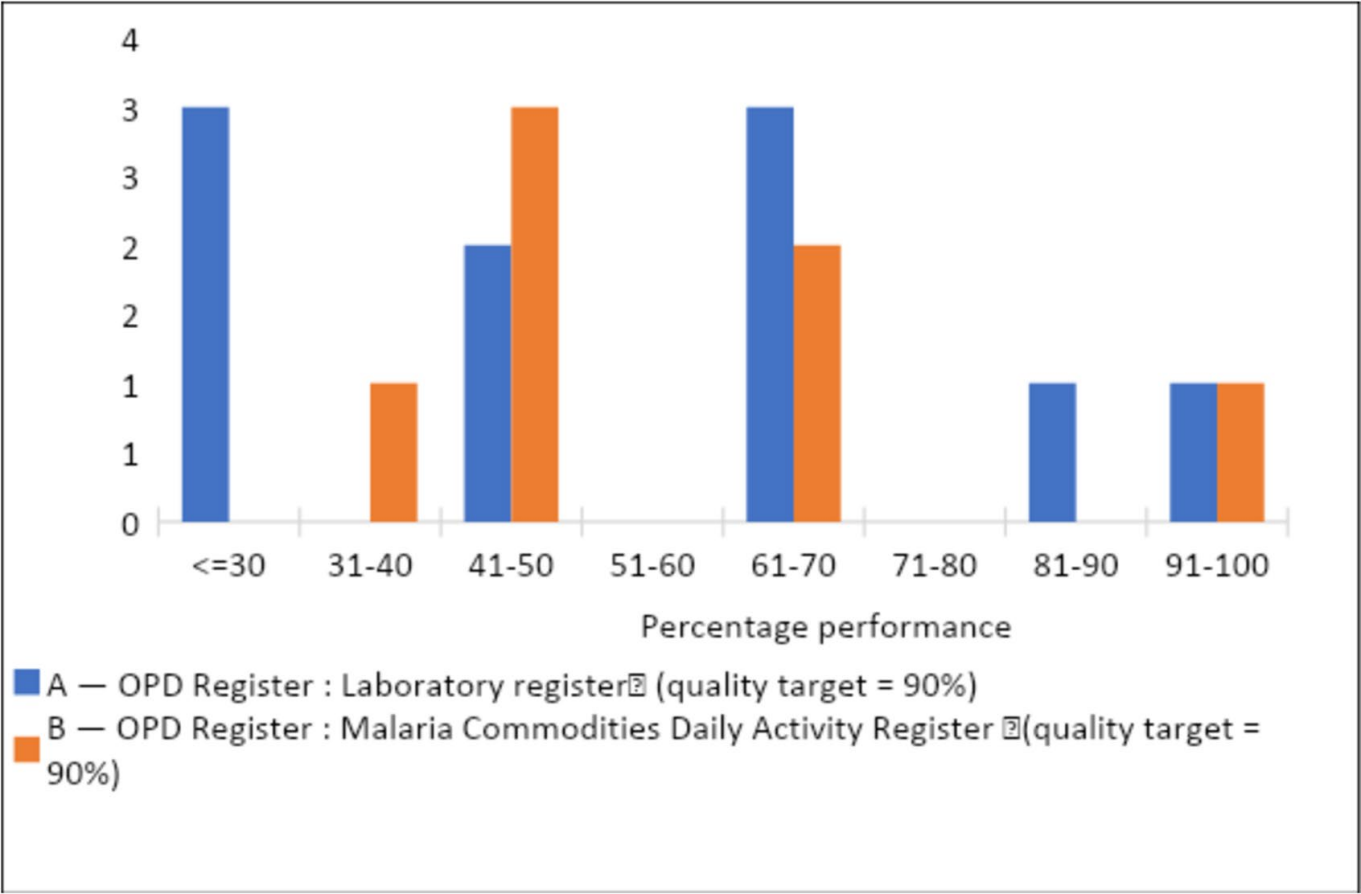
Cross Checks A and B—OPD register (MOH 204 A and B) vs Laboratory Register (MOH 240) and Daily Activity Register (MOH 645) in Nandi County, March–May 2021. *OPD* Outpatient Department

On reporting of confirmed malaria cases, over-reporting (Verification Factor (VF) of ≤ 70%) was noted in three facilities in both the facility summary tool and KHIS. Under-reporting by over 30 percentage points was noted in one facility (Fig. 8). Arithmetic errors contributed to most of the reasons for discrepancies noted, followed by transcription errors in the number of suspected, confirmed, and treated malaria cases by the health facilities (Fig. 9). Matching crosschecks conducted revealed that when ten confirmed malaria cases as recorded in the outpatient register were sampled, three facilities had less than 30% of the same cases reflecting in the laboratory register. Around 31–40% of the cases could not be traced in the malaria commodities daily activity register in one facility while the records were matching (91–100%) in one facility (Fig. 10). On the systems’ ability to generate quality data, we found written guidelines, up-to-date display of malaria cases, and chart of disease incidence were only available in 11% of the health facilities. Patient history could only be traced in 22% of the health facilities. Only 22% of the facilities had established targets for malaria control and prevention.

### Facility laboratory quality assessment

The EQA assessment tool was administered to 10 facilities, with eight of them utilizing both Microscopy and RDT procedures for malaria diagnosis. Kapsabet County Referral Hospital and Meteitei Sub-County Hospital exclusively conducted malaria diagnosis using microscopy. In four of the assessed facilities, clinical staff other than laboratory staff performed malaria diagnosis using RDTs. Among these facilities, three had RDT standard operating protocols (SOPs) available and displayed at the working area, while three had SOPs but did not display them. Two facilities had no SOPs at all.

Sub-county and higher-level facilities demonstrated better staffing ratios and a diverse composition of laboratory officers and technologists. Level three facilities had an average of three technologist cadre officers and no laboratory officer cadre. Level two facilities had an average of one laboratory technologist cadre officer, with most facilities reporting staffing shortages. Among the assessed facilities, nine out of ten had at least one laboratory personnel trained in Malaria Microscopy Refresher Training, and eight out of ten reported that malaria testing was conducted and reported solely by the microscopy trained laboratory officers.

All assessed facilities had at least one functional microscope with standard maintenance kits. Routine microscope cleaning maintenance was reported by all facilities, although only two kept logs to verify maintenance. Standard microscopy SOPs were present in all facilities, with six out of ten having them displayed. Documentation of microscopy internal quality control (IQC) programmes was observed in only three out of ten facilities, while two other facilities conducted the IQC programme but did not document it. Three facilities stored malaria slides for up to one week for re-reading. Participation in the national malaria MOH (KNEQAS) Proficiency Testing (MOH PT) scheme was reported by only two facilities, with one unable to provide the MOH PT records for verification. Turnaround time for test results was documented in the laboratory register by only two facilities, with average turnaround times of 20 and 30 min in the last week, respectively.

It was observed that four out of ten assessed facilities had the recent version of the MOH Parasitological Diagnosis guidelines, while three out of ten had the recent national guideline on diagnosis, treatment, and prevention of malaria in Kenya. Printed lab request forms were available in four out of ten facilities, while most facilities utilized the patient's booklet for requesting a laboratory test. All facilities had a dedicated column or separate register for recording blood slide results.

## Discussion

Malaria cases upsurge in Nandi County showed seasonality trend coinciding with during or after the long or short rainy seasons and although there was an increase in cases, the investigation findings did not though reveal the presence of an active outbreak during the investigation period. Retrospective case visualization and analysis indicate alert threshold levels reached, but not reaching action thresholds indicative of an active outbreak in a majority of the facilities reviewed. However, in Tinderet and Aldai sub-county hospitals, malaria cases did exceed both the alert and action thresholds at certain times previously during the year under review, suggesting potential occurrence of outbreaks. Facilities in the lowland areas of Aldai and Tinderet sub counties bordering the stable malaria endemic lake region revealed notably high facility total positivity rates despite testing low number of persons. Importation of malaria parasites to the highland as well as travel to and from the highlands as a source of new infections amongst persons living and working there could explain the high incidence and prevalence of malaria in these sub-counties due their proximity to the lake endemic region as postulated by Arness et al. in a study in this region [13]. The key informant interviews reveal insufficient exposure to the Kenya Malaria Management guidelines and policy during the review period. This was evidenced by the majority of healthcare workers having over three years’ experience but notably less than 35% being familiar with the current national malaria guidelines and policies. In addition, capacity development attributes reveal insufficient current training in critical areas such as malaria case management and surveillance. Previous authors have shown that insufficient skills in core competencies of malaria management could be barrier facilitators that lead to poor quality data generation and limited interaction between data producers and data use further widening the disconnect in data demand and use [14, 15].

Data-driven decision-making amongst national malaria control programmes in Africa is critical for the efficient use of resources in an often increasingly stratified malaria burden. Previous studies underpin the benefit of improved data quality reports for an enhanced malaria surveillance programme with data driven decision making critical for efficient use of resources [16]. In regard to the quality of the data generated, the analysis showed a majority of the facilities assessed submitted the monthly reports on time with previous studies conducted showing clear benefits of improved data quality reports by increased reporting in enhanced malaria surveillance programmes [16]. However, a majority of over 75% of the facilities reviewed did regularly conduct data review meetings with only one quarter of these facilities utilizing their routine data for malaria data analysis. Additionally, significant data quality issues such as discrepancies between source health facility documents, monthly summaries and KHIS data were noted.

The majority of the facilities either over reported or under reported suspected and confirmed malaria cases in their monthly summaries or KHIS, additionally some facilities reviewed had incompleteness of the monthly reports and primary source documents. Incomplete data gives rise to chances of a number of reported cases of the suspected malaria going unconfirmed with studies like Zunda et al. in Lusaka findings agreeing that despite the improved reporting and testing rates confirmed cases still remain low [16]. Under reporting and over reporting of suspected and confirmed malaria cases in the facility registers and monthly summaries puts accurate estimation of the true burden of malaria at risk. Accurate malaria data is key to knowing the true malaria burden, enabling hot spots identification and early outbreak detection, and enabling wise resource and intervention allocation [16]. While routine surveillance data has proven essential for malaria control, its quality limits its utility [17–19], further research work has shown focusing on malaria data quality and access improvement could prove effective for surveillance as a core intervention in malaria control [20].

The findings of the investigation noted the minimal or lack of IQC programmes in most facilities assessed with the majority of laboratories conducting microscopy not enrolled in the Ministry of Health proficiency test (PT) Programme, noting this could have an effect on the quality of tests conducted. The major RDT stock outs experienced by the majority of the facilities could have an impact on the adherence to guidelines of testing all patients with suspected malaria cases before treating them to prevent overuse and misuse of anti-malarials. Increasing the number of microscopists, with regular training of laboratory officers in microscopy refresher training at the lower level three and below facilities could improve the quality of tests conducted at these facilities with previous studies conducted showing microscopists who had recently completed refresher training and worked in a QA-pilot facility performed the best overall [17]. National EQA proficiency test programmes provide an important method to improve the quality of malarial microscopic diagnosis services, enrolment of the facilities undertaking microscopy in the county would address deficiencies like poor competency, poor infrastructure and work practices that require alteration to ensure the quality of tests conducted in the county [18]. Use of non-laboratory cadre in health facilities to conduct malaria diagnosis using RDTs holds an advantage in addressing the gaps in adherence to the test and treat management and treatment guidelines by the division of national malaria programme in malaria diagnosis [19]. The investigation highlights the staffing inadequacies with limited trained microscopists, further complicated by the operational complexities like erratic electricity and water supply, this challenges have been shown to be effectively managed with the use of RDTs for early diagnosis and prompt treatment of cases with effective anti-malarial drugs [20]. Furthermore, quality controlled RDTs when used correctly have been shown to give good results comparable to light microscopy under routine use [21]

In conclusion, there was a malaria cases upsurge that coincided with the after-long rains peak seasons as expected with increased projections of seasonality. The analysis revealed substantial data quality problems in most reviewed health facilities, compromising the reliability of malaria case burden estimates for the county. The analysis also noted that RDTs had a higher test positivity rate than microscopy despite more tests being conducted using microscopy. Further studies on the quality of tests conducted would shed more light on this discrepancy. Enhanced IQC programmes and enrolment of more facilities to the National EQA programme would improve the quality and outcomes of malaria diagnostic tests conducted.

To strengthen malaria surveillance in the county, it is recommended the county and sub-county teams cascade down trainings on malaria surveillance, encourage frequent facility malaria data review meetings and sub-county teams should conduct DHIS 2 malaria data analysis and disseminate the findings in a timely manner to the appropriate stakeholders. To strengthen case detection and management, it is recommended the county malaria coordinator, the county laboratory head, the county laboratory quality assurance head and the sub-county trainers of trainers plan and offer mentorship on microscopy and IQC to lower level facilities. To strengthen data management and quality, it is recommended timely sensitizations on new revised data capture registers and summaries to lower level facilities. This study was not without limitations; there could have been discrepancy in the calculated thresholds since the national EPR dashboard surveillance thresholds data compared to the county malaria surveillance thresholds data lacked complete 2016 data to complete the 5-year period that was required to calculate alert and action thresholds. This was mitigated by selecting facilities for assessment within the county sentinel sites, facility sub-county teams kept a record of the calculated weekly thresholds for the assessment period.

## Data Availability

The data sets informing the results and conclusion of this investigation are available upon request to the corresponding author.

## Abbreviations

MOH: Ministry of Health
DQA: Data quality assessment
MrDQA: Malaria Routine Data Quality Assessment tool
TPR: Test positivity rate
RDT: Rapid diagnostic test
EQA: External quality assurance
IQC: Internal quality control
KHIS: Kenya Health Information System
PAPfPR2-10: Population-Adjusted Parasite Prevalence of Plasmodium falciparum at 2 years of age
IQR: Interquartile range
LLIN: Long-lasting insecticide nets
IRS: Insecticide residual spraying
LSM: Larvicidal Source Management
SBC: Social behavior change
SOP: Standard operating protocols
DHIS2: District Health Information System 2
KNEQAS: Kenya National External Quality Assessment Scheme
PT: Proficiency testing
QA: Quality assurance
EPR: Epidemic preparedness and response
